# Impact of low-level sex chromosome mosaicism (<10%) on clinical outcomes of first fresh embryo transfer: a propensity score-matched retrospective cohort study

**DOI:** 10.3389/fendo.2026.1873048

**Published:** 2026-07-16

**Authors:** Keng Feng, HaiWen Wei, YuDi Luo, DeRong Li, Xiang Li, Chun Yang, LiangYing Zhou, ShaYan Tan, LingLing Zhu

**Affiliations:** 1 Reproductive Medicine Center, Yulin Maternal and Child Health Hospital, Yulin, Guangxi, China; 2 Department of Gynecology, Beiliu Second People’s Hospital, Yulin, Guangxi, China; 3 Department of Traditional Chinese Medicine, Yulin Maternal and Child Health Hospital, Yulin, Guangxi, China

**Keywords:** assisted reproductive technology, clinical pregnancy rate, infertility, karyotype, live birth rate, propensity score matching, sex chromosome mosaicism

## Abstract

**Background:**

Low-level sex chromosome mosaicism (SCM; abnormal cell proportion <10%) is frequently detected during preconception karyotype screening, yet its clinical relevance in assisted reproductive technology (ART) remains controversial. This study aimed to determine whether low-level SCM affects clinical outcomes in the first fresh embryo transfer cycle.

**Methods:**

This single-center retrospective cohort study included 3,216 infertile patients undergoing their first *in vitro* fertilization (IVF) or intracytoplasmic sperm injection and embryo transfer (ICSI-ET) cycle between January 2015 and December 2024 (261 in the SCM group and 2,955 in the control group). A 1:4 nearest-neighbor propensity score matching (PSM) was performed to balance comprehensive baseline demographic, ovarian reserve, and stimulation parameters. After matching, 221 SCM patients were paired with 881 controls. The primary outcome was live birth rate; secondary outcomes included clinical pregnancy rate and early miscarriage rate. Multivariable logistic regression was applied to the entire cohort to calculate adjusted odds ratios (aORs) and 95% confidence intervals (CIs), with age-stratified analysis to assess interaction effects.

**Results:**

After PSM, the vast majority of covariates achieved excellent balance with standardized mean differences (SMD) < 0.1. No significant differences were observed between the SCM and control groups in live birth rate (34.39% vs. 40.75%), clinical pregnancy rate (45.25% vs. 49.72%), or early miscarriage rate (20.00% vs. 16.44%). Multivariable logistic regression showed no independent association between SCM status and live birth (aOR 0.890, 95% CI 0.633–1.252, P = 0.503) or clinical pregnancy (aOR 0.915, 95% CI 0.661–1.266, P = 0.591). Female age was the only consistently significant predictor: each additional year reduced live birth odds by 12.8% (aOR 0.872, 95% CI 0.824–0.923, P < 0.001) and clinical pregnancy odds by 9.7% (aOR 0.903, 95% CI 0.856–0.952, P < 0.001). Age-stratified analysis across four subgroups (<35, 35–37, 38–40, and >40 years) demonstrated consistent null results, with absolute differences remaining below 5 percentage points and parallel declining trends across strata.

**Conclusion:**

Low-level sex chromosome mosaicism (<10%) does not significantly affect live birth rate, clinical pregnancy rate, or early miscarriage rate in the first fresh ART transfer cycle, with consistent null findings across all age strata. Female age remains the predominant determinant of ART outcomes. These findings provide robust evidence-based guidance for clinical management and genetic counseling of infertile patients with low-level sex chromosome mosaicism.

## 1 Introduction

Sex chromosome mosaicism (SCM) is defined by the coexistence of two or more distinct sex chromosome karyotypes within a single individual. The major karyotypes of female SCM include 45,X/46,XX, 47,XXX/46,XX, and 45,X/46,XY, each exhibiting distinct phenotypic and reproductive consequences (1). Epidemiological studies indicate that the prevalence of sex chromosome mosaicism in the general population ranges from 0.1% to 1.0%, whereas the detection rate in infertile populations undergoing assisted reproductive technology (ART) evaluation can reach 1%–2% (2, 3). With the routine application of chromosomal karyotype analysis in infertility workups, an increasing number of low-level sex chromosome mosaicism cases—characterized by an abnormal cell line proportion below 10%—have been identified (4). However, the clinical significance of such low-level mosaicism, particularly regarding reproductive potential, remains incompletely characterized.

The relationship between female sex chromosome mosaicism and fertility has long been debated. Early investigations primarily focused on clinically apparent Turner syndrome (TS) patients, demonstrating that 45,X/46,XX mosaic TS patients achieved natural pregnancy rates of approximately 15%–48%, significantly higher than the 1%–3% observed in non-mosaic 45,X TS patients (5, 6). Nevertheless, these studies were largely confined to phenotypically evident TS cohorts, leaving the fertility potential of women with low-level sex chromosome mosaicism without overt clinical stigmata poorly understood. In 2001, Sonntag et al. (7) compared 20 low-level sex chromosome mosaic women (≤10%) with 20 chromosomally normal controls across 38 intracytoplasmic sperm injection (ICSI) cycles, reporting no significant effect of mosaicism status on ovarian response, fertilization rate, embryo quality, or pregnancy outcomes. However, this seminal study was constrained by a small sample size and the absence of rigorous confounding factor control, thereby limiting the generalizability and statistical robustness of its conclusions.

The reproductive impact of different mosaic karyotypes exhibits substantial biological heterogeneity, underscoring the need for karyotype-specific analyses. Women with 47,XXX/46,XX mosaicism demonstrate markedly milder clinical phenotypes compared with 45,X monosomy, with spontaneous puberty rates of 83%–88%, natural pregnancy rates of approximately 69%, and healthy live birth rates reaching 71.4% (8, 9). This phenotypic attenuation may be attributed to gene dosage compensation mediated by X-chromosome inactivation (XCI) in the 47,XXX cell line, which partially mitigates the adverse effects of X-linked gene haploinsufficiency on ovarian function (10). Conversely, most women with 45,X/46,XY mosaicism lose autonomous fertility due to complete or mixed gonadal dysgenesis, face elevated risks of gonadal germ cell tumors, and typically require oocyte donation to achieve pregnancy (11). These divergent outcomes emphasize that aggregating heterogeneous mosaic karyotypes into a single “mosaic” category may obscure biologically meaningful differences and compromise the interpretability of pooled analyses.

From a molecular mechanistic perspective, the X chromosome harbors numerous genes essential for ovarian function, including BMP15, GDF9, and FMR1. These genes regulate oocyte development and follicular recruitment through the transforming growth factor-β (TGF-β) signaling pathway, and their dosage abnormalities may significantly impair ovarian reserve (12, 13). In 45,X/46,XX mosaic individuals, the 45,X cell line retains full transcriptional activity of its single X chromosome, whereas the 46,XX cell line undergoes random XCI through XIST-mediated epigenetic silencing. As the proportion of 45,X cells increases, the cumulative effect of functionally haploid X chromosomes may progressively compromise ovarian reserve; however, when mosaicism proportions remain low (<10%), normal cell lines may preserve overall ovarian function through compensatory mechanisms (14, 15). The precise threshold at which mosaicism proportion transitions from clinically inconsequential to functionally detrimental remains undefined, representing a critical knowledge gap in reproductive endocrinology.

The present study was designed to address these limitations by employing a large-scale retrospective cohort design combined with 1:4 propensity score matching (PSM) to rigorously control for confounding factors. We systematically evaluated the impact of low-level sex chromosome mosaicism (<10%) on clinical outcomes during the first fresh ART transfer cycle. Through age-stratified analyses and multivariable regression modeling, we further investigated potential interaction effects between mosaicism status and female age, aiming to provide evidence-based guidance for fertility counseling and clinical management in women with sex chromosome mosaicism.

## 2 Materials and methods

### 2.1 Study design and ethics

This single-center retrospective cohort study included infertile patients who underwent their first assisted reproductive technology (ART) treatment at the Reproductive Medicine Center of Yulin Maternal and Child Health Hospital between January 2015 and December 2024. The study protocol was approved by the Institutional Review Board of Yulin Maternal and Child Health Hospital. Informed consent was waived due to the retrospective nature of the study and the use of de-identified clinical data. All procedures adhered to the Declaration of Helsinki (2013 revision) and the Strengthening the Reporting of Observational Studies in Epidemiology (STROBE) guidelines for observational studies (16).

### 2.2 Inclusion and exclusion criteria

Inclusion criteria were as follows: (1) female age 20–45 years; (2) first *in vitro* fertilization (IVF) or intracytoplasmic sperm injection (ICSI) cycle at our center; (3) complete peripheral blood chromosomal karyotype analysis report; (4) complete cycle parameters and outcome records; and (5) fresh embryo transfer cycle.

Exclusion criteria were as follows: (1) high-level mosaicism (abnormal cell proportion ≥10%), autosomal mosaicism, or chromosomal structural rearrangements (e.g., balanced translocations, inversions); (2) severe male factor infertility requiring testicular sperm extraction or donor sperm; (3) severe endometriosis (American Society for Reproductive Medicine [ASRM] stage III–IV); (4) recurrent pregnancy loss (≥2 consecutive spontaneous abortions); (5) known uterine malformations, endometrial lesions, or uterine fibroids affecting uterine cavity morphology; (6) oocyte donation cycles; and (7) concurrent severe medical conditions (e.g., severe cardiovascular disease, uncontrolled diabetes mellitus, or thyroid disease).

### 2.3 Group definitions

Based on peripheral blood chromosomal karyotype analysis results, patients were categorized into two groups: the low-level sex chromosome mosaicism group (abnormal cell proportion <10%; karyotypes including 45,X/46,XX, 47,XXX/46,XX, and 45,X/46,XX/47,XXX) and the chromosomally normal control group (46,XX). All karyotype analyses employed standard G-banding techniques, independently interpreted by two senior cytogenetic technologists, with a minimum of 100 metaphase cells counted per patient. An abnormal cell proportion <10% was defined as low-level mosaicism, referencing the seminal study by Sonntag et al. (7) and conventional classification standards in Turner syndrome research.

### 2.4 Outcome measures

The primary outcome was the live birth rate (LBR) in the first fresh transfer cycle, defined as the proportion of transfer cycles resulting in delivery of a viable neonate at ≥28 weeks of gestation. The formula was: LBR = (number of live birth cycles/total number of transfer cycles) × 100%. We acknowledge that this ≥28-week threshold reflects Chinese clinical standards and deviates from the international standard of ≥22 or ≥24 weeks used by ICMART and most European and North American ART registries. This deviation limits direct cross-study comparability and should be considered when interpreting our findings in the context of international literature.

Secondary outcomes included: (1) Clinical pregnancy rate (CPR): the number of clinical pregnancy cycles divided by the number of transfer cycles, multiplied by 100%. Clinical pregnancy was defined as the presence of a gestational sac with fetal cardiac activity on transvaginal ultrasound at 35 days post-transfer. (2) Early miscarriage rate (EMR): the number of early miscarriage cycles divided by the number of clinical pregnancy cycles, multiplied by 100%. Early miscarriage was defined as spontaneous abortion before 12 weeks of gestation, excluding ectopic pregnancies and biochemical pregnancies.

### 2.5 Study design and sample size considerations

This study was designed as a retrospective propensity score-matched cohort study. A retrospective sample size calculation was performed based on published data from non-preimplantation genetic testing (PGT) IVF/ICSI literature. It should be specifically noted that peer-reviewed publications reporting live birth rates among patients with low-level sex chromosome mosaicism (<10%) following conventional IVF/ICSI (non-PGT) are extremely limited. Consequently, the baseline live birth rate for the mosaicism group was derived primarily from the observed data of our institutional cohort, whereas the baseline live birth rate for the normal karyotype control group was referenced from published literature on general IVF populations.

The parameters for sample size estimation were as follows:

Primary outcome: Live birth rate (LBR) per embryo transfer cycle.

Baseline live birth rate for the normal karyotype control group (non-PGT IVF population): Approximately 40–43.7%. Insogna et al. (2021) reported a live birth rate of 43.7% for frozen non-PGT embryo transfer (17), and the live birth rate for general IVF/ICSI populations has been documented in the range of 40–50% (18).

Baseline live birth rate for patients with low-level sex chromosome mosaicism (non-PGT): The observed live birth rate in our study cohort was 34.39% (76/221). Given the current absence of large comparative studies specifically reporting live birth rates among patients with low-level sex chromosome mosaicism (<10%) following conventional IVF/ICSI (non-PGT), this baseline rate was derived directly from our institutional cohort during 2015–2023.

Clinically meaningful effect size: An absolute reduction in live birth rate of 10% (from 40% to 30%), corresponding to a relative reduction of 25%.

Type I error (α): 0.05 (two-sided).

Desired power (1–β): 0.80.

Statistical test: Two-proportion z-test for comparing live birth rates between two independent groups.

Based on the aforementioned parameters, G*Power 3.1 software (Heinrich Heine University, Düsseldorf, Germany) estimated that a sample size of approximately 120 patients per group would be required (120 in the mosaicism group and 480 in the control group under a 1:4 matching ratio) to achieve 80% power for detecting a 10% absolute difference in live birth rate. The present study ultimately enrolled 221 patients in the mosaicism group, exceeding the target sample size for the exposed group and thereby providing adequate statistical power for detecting moderate-to-large effects (odds ratio [OR] ≤ 0.50 or ≥ 2.00).

### 2.6 Propensity score matching

To control for the effects of confounding factors on outcomes, a 1:4 propensity score matching (PSM) method was employed. Covariates included in the propensity score model were: female age, male age, body mass index (BMI), duration of infertility, basal follicle-stimulating hormone (FSH), basal luteinizing hormone (LH), basal prolactin, basal estradiol, basal testosterone, basal progesterone, anti-Müllerian hormone (AMH), antral follicle count (AFC), stimulation protocol (antagonist protocol/long protocol/mild stimulation protocol), fertilization method (IVF/ICSI), total gonadotropin (Gn) dose, Gn administration days, initial Gn dose, trigger-day estradiol, trigger-day LH, trigger-day progesterone, endometrial thickness, number of embryos transferred, and embryo type (cleavage stage/blastocyst). Nearest neighbor matching was performed with a caliper of 0.02. Post-matching balance was assessed using standardized mean differences (SMD), with SMD < 0.1 considered excellent balance and SMD 0.1–0.25 considered acceptable (19).

### 2.7 Statistical analysis

Statistical analyses were performed using SPSS 26.0 (IBM Corp., Armonk, NY, USA) and R 4.3.1 (R Foundation for Statistical Computing, Vienna, Austria). Normally distributed continuous variables were expressed as mean ± standard deviation (SD) and compared between groups using independent-sample t-tests. Categorical variables were expressed as frequencies (percentages) [n (%)] and compared using χ² tests or Fisher’s exact tests. Post-matching outcome comparisons were conducted using McNemar’s tests or conditional logistic regression to account for the matched design. Multivariable analyses were performed using logistic regression models incorporating female age, male age, BMI, duration of infertility, basal FSH, AMH, AFC, and mosaicism status. Age-stratified analysis was conducted across four subgroups: < 35 years, 35–37 years, 38–40 years, and > 40 years. All tests were two-tailed, with a P-value < 0.05 considered statistically significant. Missing data were handled using multiple imputation, with sensitivity analyses performed using complete case analysis.

### 2.8 Funding

This study was funded by the School-Level Research Project of Youjiang Medical University for Nationalities (Contract No. yy2025ky084).

## 3 Results

### 3.1 Study flow

**Figure 1:**
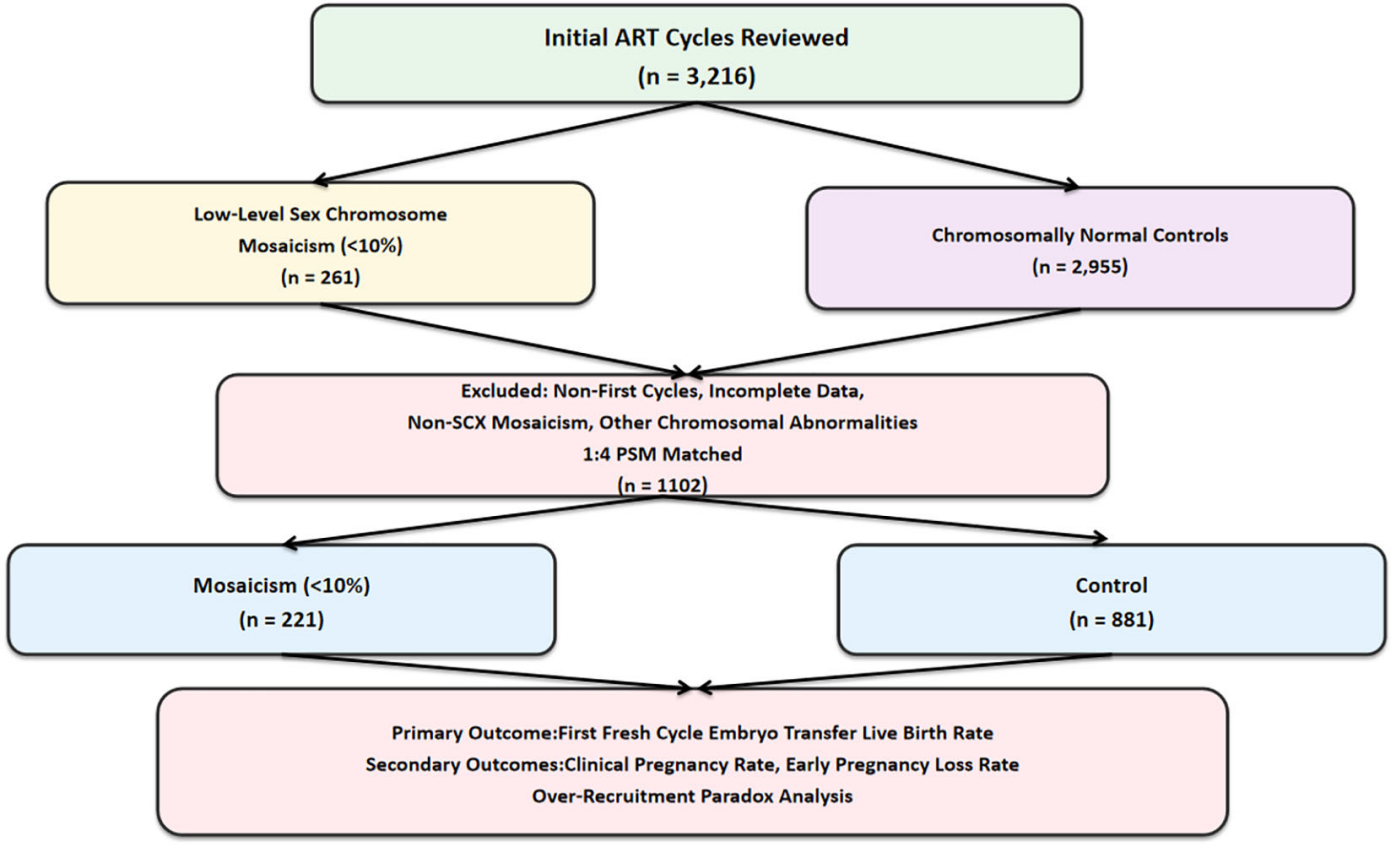
CONSORT flow diagram of patient selection and propensity score matching. PSM, Propensity Score Matching; SCX, Sex Chromosome; ART, Assisted Reproductive Technology.

The study flow is illustrated in **Figure 1**. A total of 3,216 patients were initially included in the analysis (261 in the mosaic group and 2,955 in the control group). After applying exclusion criteria and 1:4 propensity score matching, 221 mosaic patients were successfully matched with 881 control patients, constituting the final matched cohort for outcome analysis.

### 3.2 Baseline characteristics and propensity score matching performance

Before matching, the mosaic group (n = 261) and the control group (n = 2,955) exhibited substantial differences across multiple baseline characteristics. The mosaic group was characterized by significantly older female age (36.3 ± 4.1 versus 32.5 ± 4.6 years) and older male age (37.5 ± 5.0 versus 34.5 ± 4.9 years). With respect to ovarian reserve markers, the mosaic group demonstrated significantly lower anti-Müllerian hormone (AMH) levels (2.99 ± 2.64 versus 3.63 ± 2.54 ng/mL) and a reduced antral follicle count (14 ± 8 versus 17 ± 8).

After 1:4 propensity score matching, 221 matched sets were obtained, comprising 221 patients in the mosaic group and 881 patients in the control group. The vast majority of covariates achieved excellent balance with standardized mean differences (SMD) < 0.1. Two covariates exhibited minimal residual imbalance just above the conventional 0.1 threshold: trigger-day luteinizing hormone (LH) level (SMD = 0.108) and stimulation protocol (SMD = 0.121); both values remained well below the lenient threshold of 0.2, confirming acceptable between-group comparability. Overall, the matched cohort demonstrated satisfactory covariate balance (**Table 1**). The Love plot of standardized mean differences comprehensively illustrated the balance of all covariates before and after matching.

**Table 1:** Baseline characteristics before and after propensity score matching.

Characteristic	Before matching			After matching		
	Normol N = 2,955	Mosaicism N = 261	SMD^†^	normol N = 881	Mosaicism N = 221	SMD^†^
Female Age, Mean ± SD	32.5 ± 4.6	36.3 ± 4.1	0.939	35.4 ± 4.0	36.3 ± 4.1	0.070
Male Age, Mean ± SD	34.5 ± 4.9	37.5 ± 5.0	0.606	36.8 ± 5.0	37.5 ± 4.9	0.046
BMI Body Mass Index, Mean ± SD	22.2 ± 3.2	23.0 ± 2.9	0.281	22.77 ± 3.28	22.95 ± 2.93	0.033
Duration of Infertility, Mean ± SD	4.2 ± 3.0	4.8 ± 3.8	0.161	4.7 ± 3.6	4.9 ± 3.9	0.046
Basal FSH, Mean ± SD	6.05 ± 2.10	6.41 ± 2.70	0.133	6.04 ± 2.12	6.20 ± 2.39	0.061
Basal LH, Mean ± SD	3.68 ± 2.40	3.87 ± 6.22	0.031	3.45 ± 2.39	3.67 ± 5.65	0.052
Basal Prolactin, Mean ± SD	22 ± 39	24 ± 48	0.026	22 ± 42	21 ± 20	-0.014
Basal Estradiol, Mean ± SD	32 ± 16	117 ± 412	0.206	35 ± 17	39 ± 34	0.008
Basal Testosterone, Mean ± SD	0.35 ± 0.70	0.34 ± 0.37	-0.021	0.34 ± 0.27	0.34 ± 0.38	0.010
Basal Progesterone, Mean ± SD	0.34 ± 0.30	0.67 ± 3.09	0.106	0.36 ± 0.34	0.39 ± 1.03	0.009
Basal AMH, Mean ± SD	3.63 ± 2.54	2.99 ± 2.64	-0.242	3.15 ± 2.47	3.03 ± 2.68	-0.022
Total Antral Follicle Count AFC, Mean ± SD	17 ± 8	14 ± 8	-0.380	14 ± 7	14 ± 8	0.002
Endometrial Thickness on Final Ultrasound, Mean ± SD	10.63 ± 1.68	10.31 ± 2.53	-0.127	10.66 ± 1.84	10.37 ± 2.53	-0.078
Total Gonadotropin Dose, Mean ± SD	1,739 ± 1,025	2,285 ± 710	0.769	2,306 ± 839	2,307 ± 719	-0.052
Total Days of Gonadotropin Administration, Mean ± SD	9.77 ± 2.71	10.21 ± 2.02	0.219	10.48 ± 2.03	10.30 ± 2.03	-0.055
Initial Gonadotropin Dose, Mean ± SD	159 ± 67	216 ± 56	1.03	209 ± 58	216 ± 55	-0.001
Final Ultrasound Estradiol Level, Mean ± SD	1,772 ± 1,274	2,282 ± 1,266	0.403	2,208 ± 1,147	2,297 ± 1,271	0.041
Final Ultrasound LH Level, Mean ± SD	3.6 ± 4.7	1.6 ± 1.5	-1.32	1.40 ± 1.85	1.55 ± 1.44	0.108
Final Ultrasound Progesterone Level, Mean ± SD	0.68 ± 0.36	0.64 ± 0.36	-0.107	0.63 ± 0.31	0.65 ± 0.36	0.023
Total Oocytes Retrieved, Mean ± SD	9.2 ± 5.2	10.4 ± 6.6	0.176	10.6 ± 5.2	10.4 ± 6.6	-0.025
Embryo Transfer Day, Mean ± SD	3.80 ± 1.03	3.87 ± 1.15	0.056	3.74 ± 0.98	3.87 ± 1.15	0.075
Stimulation protocol, n (%)						
GnRH Agonist Protocol	1,842 (62%)	134 (58%)	-0.083	515 (71%)	127 (60%)	-0.121
GnRH Antagonist Protocol	1,113 (38%)	96 (42%)	0.083	208 (29%)	86 (40%)	0.121
Obstetric history, n (%)						
NO	2,326 (79%)	149 (65%)	-0.292	483 (67%)	138 (65%)	-0.011
YES	629 (21%)	81 (35%)	0.292	240 (33%)	75 (35%)	0.011
Fertilization method, n (%)						
ICSI	1,290 (44%)	41 (18%)	-0.675	136 (19%)	39 (18%)	0.044
IVF	1,665 (56%)	189 (82%)	0.675	587 (81%)	174 (82%)	-0.044
Number of embryos transferred, n (%)						
1	1,263 (43%)	101 (44%)	0.024	329 (46%)	97 (46%)	-0.017
2	1,692 (57%)	129 (56%)	-0.024	394 (54%)	116 (54%)	0.017

^†^ SMD, Standardized Mean Difference.

### 3.3 Clinical outcomes after matching

After propensity score matching, 221 patients in the mosaic group and 881 patients in the control group were included in the outcome analysis. No significant differences were observed between the two groups in any of the evaluated reproductive outcomes (**Table 2**).

**Table 2:** ART outcomes in the propensity score-matched cohort (conditional logistic regression) data are presented as n (%) unless otherwise indicated.

Outcome	Mosaicism group (n=221)	Control group (n=881)	Matched OR (95% CI)	P value	aOR (95% CI)	P value
Clinical pregnancy	100 (45.25)	438 (49.72)	0.896 (0.661–1.214)	0.480	0.901 (0.665–1.221)	0.507
Early miscarriage	20 (20.00)	72 (16.44)	1.400 (0.657–2.985)	0.383	1.356 (0.635–2.899)	0.426
Late miscarriage	2 (2.00)	3 (0.68)	—	—	—	—
Ectopic pregnancy	2 (0.90)	4 (0.45)	—	—	—	—
Live birth	76 (34.39)	359 (40.75)	0.895 (0.647–1.239)	0.496	0.912 (0.658–1.264)	0.574

OR, odds ratio; aOR, adjusted odds ratio; CI, confidence interval.

Conditional logistic regression was used to estimate matched ORs and 95% CIs for propensity score-matched data.

The clinical pregnancy rate was 45.25% (100/221) in the mosaic group and 49.72% (438/881) in the control group [matched odds ratio (OR) 0.896, 95% confidence interval (CI) 0.661–1.214; P = 0.480]. The early miscarriage rate was 20.00% (20/100) and 16.44% (72/438), respectively (matched OR 1.40, 95% CI 0.657–2.985; P = 0.383). Late miscarriage [2.00% (2/100) versus 0.68% (3/438)] and ectopic pregnancy [0.90% (2/221) versus 0.45% (4/881)] were rare events in both groups, with insufficient numbers to permit stable odds ratio estimation.

The primary outcome—live birth rate—was 34.39% (76/221) in the mosaic group and 40.75% (359/881) in the control group (matched OR 0.895, 95% CI 0.647–1.239; P = 0.496). After further adjustment for residual confounders, the adjusted odds ratios were consistent with the matched estimates: clinical pregnancy (adjusted OR [aOR] 0.901, 95% CI 0.665–1.221; P = 0.507), early miscarriage (aOR 1.356, 95% CI 0.635–2.899; P = 0.426), and live birth (aOR 0.912, 95% CI 0.658–1.264; P = 0.574).

Collectively, these findings indicate that low-level sex chromosome mosaicism (<10%) does not increase the risk of adverse reproductive outcomes after adequate control for confounding factors.

### 3.4 Multivariable logistic regression analysis

To independently assess the impact of sex chromosome mosaicism status on ART outcomes, multivariable logistic regression models were constructed for live birth rate and clinical pregnancy rate, adjusting for female age, male age, body mass index (BMI), duration of infertility, basal follicle-stimulating hormone (FSH), AMH, antral follicle count (AFC), and mosaicism status (**Table 3**).

**Table 3:** Multivariable logistic regression analysis.

Outcome	Variable	aOR	95%_CI	P_value	Significant
Live Birth	Female Age	0.872	0.824 – 0.923	<0.001	Yes
	Male Age	1.002	0.959 – 1.046	0.942	—
	BMI	0.999	0.949 – 1.053	0.983	—
	Duration of Infertility	1.024	0.981 – 1.069	0.277	—
	Basal FSH	0.96	0.892 – 1.032	0.268	—
	Basal AMH	1.006	0.921 – 1.100	0.891	—
	Total AFC	1.004	0.974 – 1.035	0.794	—
	Mosaicism	0.89	0.633 – 1.252	0.503	—
Clinical Pregnancy	Female Age	0.903	0.856 – 0.952	<0.001	Yes
	Male Age	1.015	0.975 – 1.058	0.462	—
	BMI	1.003	0.953 – 1.054	0.921	—
	Duration of Infertility	1.014	0.973 – 1.056	0.509	—
	Basal FSH	1.004	0.941 – 1.072	0.897	—
	Basal AMH	1.033	0.946 – 1.129	0.470	—
	Total AFC	1.004	0.975 – 1.035	0.770	—
	Mosaicism	0.915	0.661 – 1.266	0.591	—

aOR, adjusted odds ratio; Cl, confidence interval; BMl, body mass index; AMH, anti-Mullerian hormone; AFC, antral follicle count.

In the live birth model, female age was the only statistically significant independent predictor (aOR = 0.872, 95% CI: 0.824–0.923, P < 0.001), with each additional year of age reducing live birth odds by approximately 12.8%. Male age, BMI, duration of infertility, basal FSH, AMH, and AFC showed no independent associations with live birth rate. Importantly, sex chromosome mosaicism status had no significant effect on live birth rate (aOR = 0.890, 95% CI: 0.633–1.252, P = 0.503), suggesting that mosaicism status itself does not alter live birth probability after comprehensive confounding adjustment.

**Figure 2:**
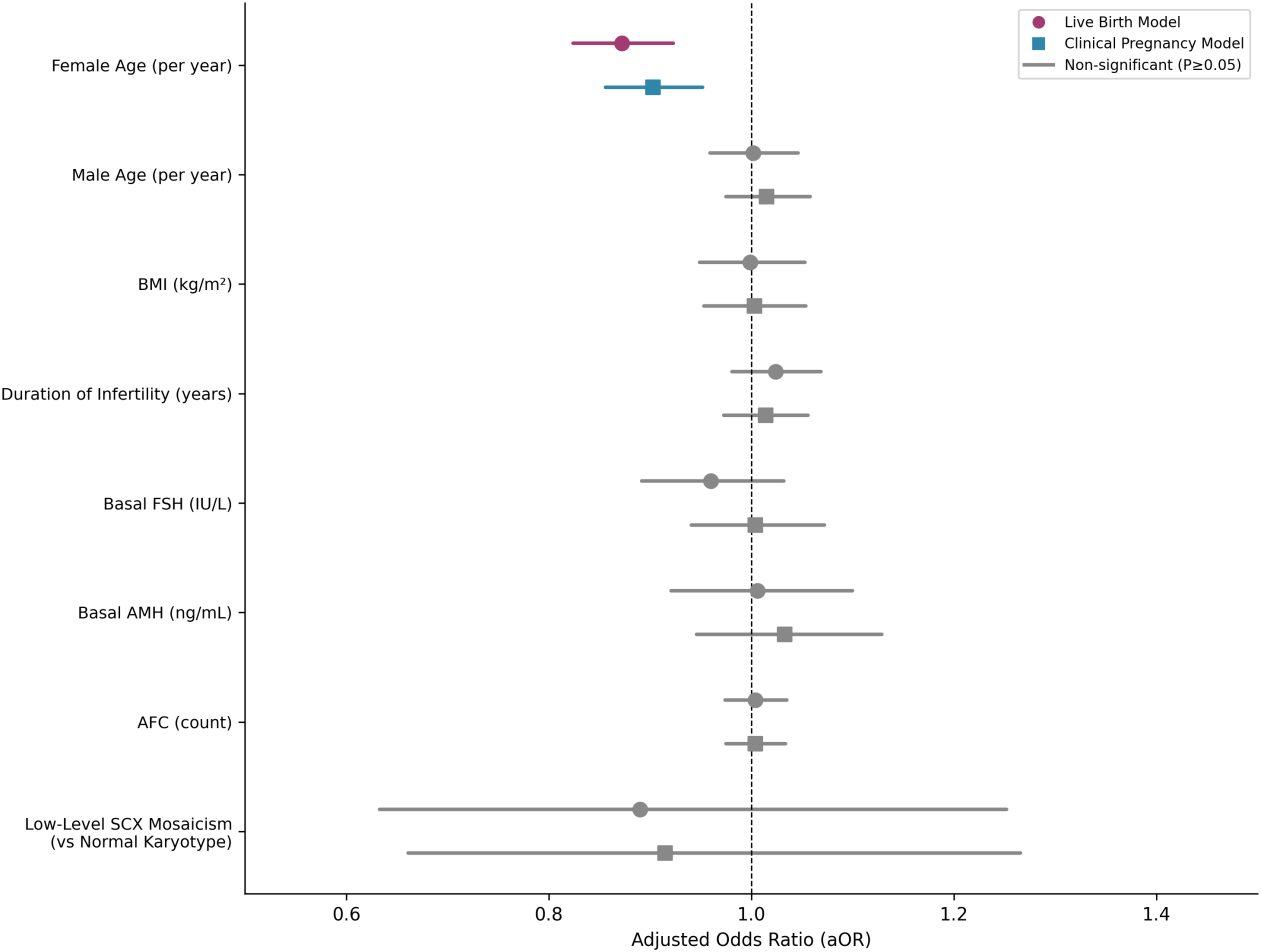
Multivariable logistic regression forest plot (primary outcomes: live birth and clinical pregnancy).

In the clinical pregnancy model, female age was similarly the only independent significant predictor (aOR = 0.903, 95% CI: 0.856–0.952, P < 0.001). Sex chromosome mosaicism status also showed no significant effect on clinical pregnancy rate (aOR = 0.915, 95% CI: 0.661–1.266, P = 0.591). These consistent results across primary and secondary outcomes further confirm that low-level sex chromosome mosaicism (<10%) exerts no independent negative effects on pregnancy establishment or maintenance in ART cycles (**Figure 2**).

### 3.5 Age-stratified analysis

**Figure 3:**
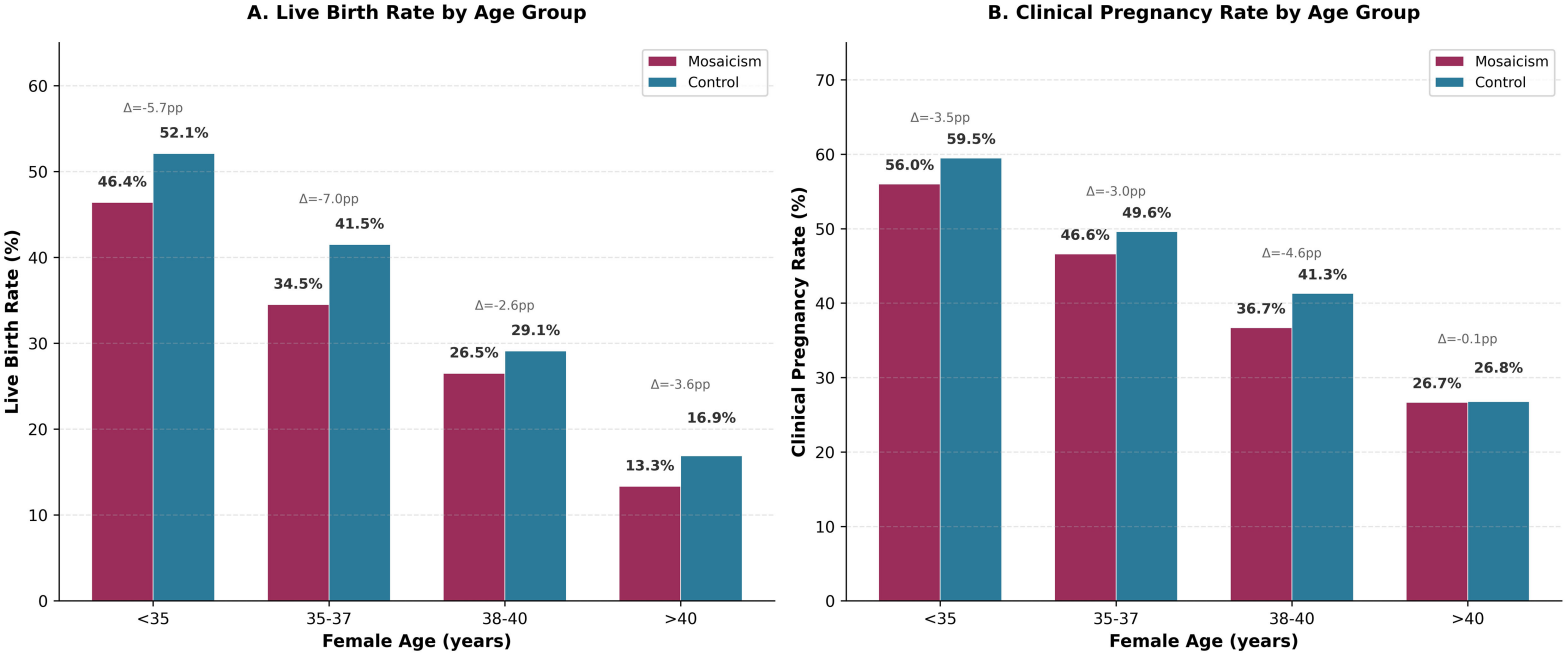
Age-stratified **(A)** live birth rate and **(B)** clinical pregnancy rate, with error bars representing 95% confidence intervals. No significant differences were observed across any age stratum.

Given that female age is the most powerful predictor of ART outcomes, age-stratified analyses were performed to examine whether the effect of mosaicism status on reproductive outcomes varied by maternal age (**Table 4**; **Figure 3**). Patients were stratified into four age categories: <35 years, 35–37 years, 38–40 years, and >40 years.

**Table 4:** Age-stratified analysis of ART outcomes in the propensity score-matched cohort.

Age group	Group	Cycles n	Clinical pregnancy n (%)	OR (95% CI)	P value	Live birth n (%)	OR (95% CI)	P value	Early miscarriage n (%)	OR (95% CI)	P value
<35 years	Mosaicism	84	47 (55.95)	0.792 (0.449–1.396)	0.419	39 (46.43)	0.760 (0.426–1.357)	0.354	7 (14.89)	1.488 (0.595–3.720)	0.444
	Control	351	209 (59.54)	—	—	183 (52.14)	—	—	22 (10.53)	—	—
35–37 years	Mosaicism	58	27 (46.55)	1.027 (0.484–2.182)	0.944	20 (34.48)	1.043 (0.467–2.330)	0.918	5 (18.52)	1.313 (0.440–3.915)	0.570
	Control	246	122 (49.59)	—	—	102 (41.46)	—	—	18 (14.75)	—	—
38–40 years	Mosaicism	49	18 (36.73)	1.384 (0.467–4.100)	0.557	13 (26.53)	0.592 (0.151–2.328)	0.453	5 (27.78)	0.917 (0.297–2.835)	1.000
	Control	213	88 (41.31)	—	—	62 (29.11)	—	—	26 (29.55)	—	—
>40 years	Mosaicism	30	8 (26.67)	1.257 (0.248–6.363)	0.782	4 (13.33)	1.186 (0.164–8.606)	0.866	3 (37.50)	1.300 (0.231–7.315)	1.000
	Control	71	19 (26.76)	—	—	12 (16.90)	—	—	6 (31.58)	—	—

Data are presented as n (%) unless otherwise indicated.

No statistically significant differences were observed between the mosaic and control groups in clinical pregnancy rate, live birth rate, or early miscarriage rate within any age subgroup. Among women <35 years, the clinical pregnancy rate was 55.95% (47/84) in the mosaic group and 59.54% (209/351) in the control group (OR 0.792, 95% CI 0.449–1.396; P = 0.419), and the live birth rate was 46.43% (39/84) and 52.14% (183/351), respectively (OR 0.760, 95% CI 0.426–1.357; P = 0.354). In the 35–37 years subgroup, the clinical pregnancy rate was 46.55% (27/58) versus 49.59% (122/246) (OR 1.027, 95% CI 0.484–2.182; P = 0.944), and the live birth rate was 34.48% (20/58) versus 41.46% (102/246) (OR 1.043, 95% CI 0.467–2.330; P = 0.918). For women aged 38–40 years, the clinical pregnancy rate was 36.73% (18/49) versus 41.31% (88/213) (OR 1.384, 95% CI 0.467–4.100; P = 0.557), and the live birth rate was 26.53% (13/49) versus 29.11% (62/213) (OR 0.592, 95% CI 0.151–2.328; P = 0.453). In the >40 years subgroup, the clinical pregnancy rate was 26.67% (8/30) versus 26.76% (19/71) (OR 1.257, 95% CI 0.248–6.363; P = 0.782), and the live birth rate was 13.33% (4/30) versus 16.90% (12/71) (OR 1.186, 95% CI 0.164–8.606; P = 0.866).

Early miscarriage rates were also comparable between groups across all age strata: 14.89% versus 10.53% (<35 years; OR 1.488, 95% CI 0.595–3.720; P = 0.444), 18.52% versus 14.75% (35–37 years; OR 1.313, 95% CI 0.440–3.915; P = 0.570), 27.78% versus 29.55% (38–40 years; OR 0.917, 95% CI 0.297–2.835; P = 1.000), and 37.50% versus 31.58% (>40 years; OR 1.300, 95% CI 0.231–7.315; P = 1.000).

Both groups exhibited the expected age-related decline in reproductive success, with live birth rates decreasing from approximately 46% and 52% in women <35 years to approximately 13% and 17% in women >40 years, respectively. The absolute between-group differences remained below 10 percentage points across all age strata and outcomes.

Taken together, these findings demonstrate that low-level sex chromosome mosaicism (<10%) does not significantly affect ART outcomes across the full spectrum of female reproductive age, including in women of advanced maternal age, although the smaller sample sizes in the >40 years subgroup warrant cautious interpretation.

Conditional logistic regression was used to estimate ORs and 95% CIs for clinical pregnancy and live birth.

Fisher exact test was used to estimate ORs and 95% CIs for early miscarriage (due to small number of events in some strata).

OR values for early miscarriage in 35–37 and 38–40 years age strata were estimated using Fisher exact test due to complete separation in conditional logistic regression.

### 3.6 Subgroup analysis

The results of the conditional logistic regression analysis for each karyotype subgroup are presented in **Table 5**. Across all three karyotype patterns, no statistically significant differences were observed between the mosaic and control groups for any of the primary outcomes. Specifically, for clinical pregnancy, the odds ratios were 0.889 (95% CI: 0.543–1.454; P = 0.639) for 45,X/46,XX, 0.897 (95% CI: 0.549–1.465; P = 0.665) for 47,XXX/46,XX, and 0.905 (95% CI: 0.483–1.696; P = 0.756) for 45,X/47,XXX/46,XX. Similarly, for live birth, the odds ratios were 0.815 (95% CI: 0.476–1.395; P = 0.456), 0.915 (95% CI: 0.547–1.532; P = 0.737), and 0.999 (95% CI: 0.510–1.956; P = 0.998), respectively. Early miscarriage rates were also comparable across all three subgroups, with odds ratios ranging from 0.782 to 1.233 (all P > 0.05).

**Table 5:** Subgroup analysis of ART outcomes by karyotype in propensity score-matched cohorts.

Karyotype	n	Clinical pregnancy n (%)	OR (95% CI)	P value	Live birth n (%)	OR (95% CI)	P value	Early miscarriage n (%)	OR (95% CI)	P value
45,X/46,XX	86/338	38 (44.19)	0.889 (0.543–1.454)	0.639	27 (31.40)	0.815 (0.476–1.395)	0.456	9 (23.68)	1.218 (0.540–2.745)	0.650
47,XXX/46,XX	84/347	40 (47.62)	0.897 (0.549–1.465)	0.665	31 (36.90)	0.915 (0.547–1.532)	0.737	8 (20.00)	1.233 (0.498–3.050)	0.841
45,X/47,XXX/46,XX	51/196	22 (43.14)	0.905 (0.483–1.696)	0.756	18 (35.29)	0.999 (0.510–1.956)	0.998	3 (13.64)	0.782 (0.207–2.951)	0.803

OR, odds ratio; CI, confidence interval. Conditional logistic regression was used to estimate ORs and 95% CIs for matched data. P values for interaction (likelihood ratio test): Clinical pregnancy P = 0.957; Live birth P = 0.856; Early miscarriage P = 0.829.

To formally test whether the effect of mosaicism on ART outcomes differed across karyotype subgroups, likelihood ratio tests for interaction were conducted. The interaction P-values were 0.957 for clinical pregnancy, 0.856 for live birth, and 0.829 for early miscarriage (**Table 5**). All interaction terms were non-significant, indicating that the association between mosaicism and ART outcomes was consistent across the three karyotype patterns.

### 3.7 *Post-hoc* statistical power analysis

Because the study was retrospective and the final sample size was determined by the available patient population rather than a formal prospective sample size calculation, a *post-hoc* statistical power analysis was conducted to evaluate the statistical power of the achieved sample size and to aid in the interpretation of the observed negative results. The power analysis was performed using the normal approximation method for comparing two proportions, implemented in Python.

The achieved sample size provided adequate power (>80%) to detect moderate to large effect sizes (OR ≤ 0.65 for clinical pregnancy and live birth, or OR ≥ 1.67 for early miscarriage). However, the power was insufficient to detect small effect sizes (OR = 0.70–0.80), which may still be clinically meaningful. For example, the observed live birth rate difference of 6.4% (40.8% versus 34.4%) corresponds to an OR of approximately 0.90, which falls in the underpowered range. Therefore, while large effects can be confidently excluded, small but clinically meaningful differences cannot be ruled out (**Table 6**).

**Table 6:** *Post-hoc* statistical power to detect specific effect sizes based on actual sample size (n = 221 vs. 881).

Primary outcome	Control rate	Mosaic rate	Power to detect OR = 0.70
Clinical Pregnancy	49.72%	45.25%	~22%
Live Birth	40.75%	34.39%	~8%
Early Miscarriage	16.44%	20.00%	~71% (OR = 2.00)

Calculations assume α = 0.05 (two-sided) using normal approximation.

## 4 Discussion

### 4.1 Principal findings

Through rigorous 1:4 propensity score matching (PSM) to control for confounding factors, this study demonstrated that low-level sex chromosome mosaicism (SCM) status, defined as an abnormal cell line proportion below 10%, had no significant effect on live birth rate (LBR), clinical pregnancy rate (CPR), or early miscarriage rate (EMR) in the first fresh assisted reproductive technology (ART) transfer cycle. This finding was consistently validated across multivariable regression models and age-stratified analyses. The principal contributions of this study are threefold: (1) adoption of a large-sample PSM design that effectively controlled for confounding biases prevalent in previous studies; (2) systematic evaluation of the interaction between low-level SCM and age through stratified analyses, thereby providing more refined evidence for clinical genetic counseling; and (3) comprehensive comparison of diverse mosaic karyotypes, enhancing the generalizability of our conclusions to the broader spectrum of female SCM.

### 4.2 Comparison with previous studies

Our findings align closely with previous small-sample exploratory studies. In 2001, Sonntag et al. (7) reported that 20 low-level sex chromosome mosaic women (≤10%) showed no significant differences compared to 20 chromosomally normal controls in ovarian response, fertilization rate, embryo quality, or pregnancy outcomes during intracytoplasmic sperm injection (ICSI) cycles. However, this seminal study was constrained by an extremely small sample size and the absence of systematic confounding factor control, thereby limiting the external validity and generalizability of its conclusions.

A recent study on ART outcomes in Turner syndrome (TS) patients with varying degrees of mosaicism provides important context for our findings. Liao et al. (19) demonstrated that low (<10%), intermediate (10–50%), and high (>50%) levels of 45,X mosaicism did not significantly affect clinical pregnancy rates, live birth rates, or cumulative live birth rates in mosaic TS patients undergoing IVF/ICSI. This finding is consistent with our core conclusion that when mosaicism proportions remain below the 10% threshold, sex chromosome mosaicism status itself does not independently influence ART outcomes. Instead, female age emerges as the dominant predictor of reproductive success.

### 4.3 Biological mechanisms

From a biological mechanistic perspective, our findings support the “cell proportion threshold” hypothesis, which posits that a detectable clinical phenotype emerges only when the abnormal cell line proportion exceeds a critical threshold.

#### 4.3.1 45,X/46,XX mosaicism

In 45,X/46,XX mosaic individuals, the functional defect of the 45,X cell line primarily stems from insufficient dosage of key genes located on the haploid X chromosome. The X chromosome harbors numerous genes essential for ovarian function, including BMP15, GDF9, and FMR1, which regulate oocyte development and follicular recruitment through the transforming growth factor-β (TGF-β) signaling pathway (12, 13). Additionally, abnormal homologous chromosome pairing during meiosis may contribute to germ cell apoptosis (20). However, when the 45,X cell line proportion is below 10%, the dominant 46,XX normal cell line can maintain overall ovarian function through several compensatory mechanisms.

First, oogonia in the normal 46,XX cell line can compensate for the functional defects of 45,X cells through clonal expansion. The primordial follicle pool is established during fetal development through mitosis and meiosis (21); when mosaicism proportions are low, the compensatory capacity of normal cell lines is sufficient to preserve adequate primordial follicle reserves. Second, X-chromosome inactivation (XCI) skewing serves as an important protective mechanism. In 46,XX cells, one X chromosome is randomly inactivated through X-inactive specific transcript (XIST)-mediated epigenetic modifications (22). Studies have demonstrated that mosaic TS women, particularly those with 46,XX or 47,XXX cell lines, exhibit significantly superior ovarian reserve markers [anti-Müllerian hormone (AMH), antral follicle count (AFC)] compared to non-mosaic 45,X patients, with measurable AMH detected in 77% of 45,X/46,XX mosaic patients versus only 10% of 45,X patients (23). Third, 45,X cells in peripheral blood of normal women accumulate linearly with age (approximately 700 per 10^6^ cells per year) (16), suggesting that low-level 45,X cells may represent an extreme variant of physiological aging rather than an independent pathological condition.

#### 4.3.2 47,XXX/46,XX mosaicism

The biological mechanisms underlying 47,XXX/46,XX mosaicism differ fundamentally from those of 45,X/46,XX mosaicism. In 47,XXX cells, the extra X chromosomes undergo XIST-mediated inactivation, resulting in two Barr bodies, a mechanism distinct from the single X chromosome inactivation observed in 45,X/46,XX mosaicism (24). However, approximately 15% of X-linked genes are “escape genes” that evade XCI silencing and continue to be expressed in triple-X cells, leading to gene product overexpression (25). Escape genes include SHOX (associated with stature), multiple neurodevelopment-related genes, and loci involved in ovarian function regulation (26). This mechanism explains why women with 47,XXX syndrome often exhibit above-average height and mild neurodevelopmental differences, while their clinical phenotypes are significantly milder than those associated with 45,X monosomy.

From a reproductive function perspective, the presence of 47,XXX cell lines can partially alleviate X-linked gene haploinsufficiency in 45,X cells through gene dosage compensation. Literature reports indicate that 45,X/47,XXX mosaic women achieve spontaneous puberty rates of 83–88%, menarche rates of approximately 57–77%, natural pregnancy rates of approximately 69%, and healthy live birth rates reaching 71.4% (8, 9). Under low-level mosaicism conditions (<10%), the gene overexpression effects of 47,XXX cells may be diluted by the majority advantage of normal 46,XX cells, thereby maintaining overall ovarian function within the clinically normal range.

#### 4.3.3 Unified theoretical framework

Despite the diverse molecular mechanisms underlying the three mosaic types, when mosaicism proportions remain below 10%, the majority advantage of normal 46,XX cells may maintain reproductive function through the following common mechanisms: (1) clonal expansion and competitive compensation of functional cell lines; (2) XIST-mediated XCI precise regulation ensuring that X-linked gene expression dosage approaches diploid normal levels; and (3) cumulative effects of escape gene overexpression or haploinsufficiency remaining below clinically detectable thresholds. This “cell proportion threshold” hypothesis provides a unified theoretical framework for understanding the benign clinical prognosis of low-level sex chromosome mosaicism.

### 4.4 Interaction between age and mosaicism status

A key finding of this study is that female age did not amplify the negative effects of low-level sex chromosome mosaicism on ART outcomes. Across four age subgroups (<35, 35–37, 38–40, and >40 years), outcome differences between the mosaic and control groups were consistently below 5 percentage points, with highly parallel declining trends and slopes. This negative finding can be understood at three levels.

I. Mechanistic Independence: Distinct Biological Pathways. Female age negatively impacts ART outcomes through several well-established mechanisms: (1) age-dependent degradation of meiotic cohesin complexes (SMC1β, SMC3, REC8, STAG3), leading to chromosome segregation errors and increased aneuploidy (27). Lister et al. reported that REC8 levels are reduced by approximately 50% in oocytes from women over 37 years of age. (2) Mitochondrial dysfunction with ATP production decreased by 30–40%, causing spindle assembly defects (28). (3) Telomere shortening, triggering DNA damage response and oocyte apoptosis (29, 30). In contrast, low-level mosaicism affects somatic X-linked gene dosage and XCI balance, rather than oocyte meiotic apparatus integrity. These represent independent biological pathways.

II. Ceiling Effect: Masking at Advanced Age. In the >40-year group, age-related oocyte aneuploidy reaches 60–80% (31), and live birth rates decline to approximately 15–20%. Even if mosaicism caused a 5–10% ovarian functional decline, this contribution would be overwhelmed by the dominant negative effect of advanced age—a phenomenon known as the “ceiling” or “floor” effect (32). Notably, in our study, the mosaic live birth rate (13.33%) was 3.57 percentage points lower than that of controls (16.90%), although this difference was not statistically significant (P = 0.866). The limited sample size in this age stratum (30 versus 71) yielded approximately 60–70% power, which was insufficient to detect effect sizes below 10%.

III. Age-Related Parallelism of Physiological Mosaicism. Machiela et al. (33) (N = 31,982) demonstrated that the incidence of X chromosome mosaicism increases with age: 0.11% under 50 years versus 0.45% over 75 years (odds ratio [OR] = 1.04 per year). Gersak et al. (34) showed that low-level mosaic premature ovarian failure (POF) women averaged 35.92 ± 3.87 years versus 26.0 ± 5.65 years for high-level mosaic women (P < 0.001). These observations suggest that low-level mosaicism may represent an “extreme manifestation” of physiological aging rather than an independent pathogenic factor.

In summary, the absence of a synergistic negative effect arises because: (1) distinct biological pathways operate independently; (2) mosaicism’s modest effect is masked by the dominant effect of age; and (3) low-level mosaicism may itself be part of age-related physiological aging. Clinicians should therefore not overemphasize the significance of low-level mosaicism in older patients, as female age remains the dominant predictor of ART outcomes.

### 4.5 Clinical implications and genetic counseling

Our data support relatively positive prognostic counseling for patients with low-level mosaicism (<10%). However, clinicians should remain vigilant regarding several important considerations: (1) Offspring chromosomal risk: TS women have elevated offspring aneuploidy rates (35, 36). Birkebaek et al. (5) reported chromosomal abnormalities in 9.4% of offspring of 45,X/46,XX mosaic women. Prenatal diagnosis is therefore recommended. (2) Mosaic proportion verification: Peripheral blood 45,X cells accumulate with age (16); repeat karyotyping or fluorescence *in situ* hybridization (FISH) is advisable for borderline cases (5–10%). Gravholt et al. (10) emphasize that peripheral blood mosaicism proportions may not accurately reflect gonadal tissue status; ovarian tissue biopsy may be considered when clinically indicated. (3) Preimplantation genetic testing for aneuploidy (PGT-A) may be considered for patients concerned about offspring risk, though cost-effectiveness requires further evaluation (37, 38).

### 4.6 Study limitations

Several limitations should be acknowledged. First, the retrospective single-center design may introduce selection and information bias. Second, unmeasured confounders, including endometrial receptivity, immune factors, and lifestyle variables, were not fully controlled. Third, the mosaic group sample size was limited (n = 261), particularly in the >40-year subgroup, which may have reduced statistical power for detecting small effect sizes. Fourth, peripheral blood karyotype analysis was performed without tissue-specific mosaicism assessment. Fifth, offspring follow-up data were not available. Sixth, only the first fresh cycle was evaluated; cumulative live birth or frozen-thawed cycle outcomes were not assessed.

We acknowledge that the 10% threshold for defining low-level mosaicism carries inherent classification uncertainty. G-banding of 100 cells has a detection limit of approximately 5–10%, meaning that patients with true mosaicism proportions of 8–9% may be stochastically misclassified into either the <10% or ≥10% category. However, several considerations suggest that this uncertainty does not invalidate our null findings. First, any misclassification at the threshold would be non-differential with respect to outcomes—it would dilute rather than exaggerate group differences. If a true biological effect existed, such misclassification would bias results toward the null (Type II error), making our observed lack of association a conservative estimate. Second, the consistent null results across all age strata and multiple analytical approaches (PSM matching, multivariable regression, and age-stratified analysis) argue against the possibility that threshold effects alone are masking a true association. Third, our control group (46,XX) by definition contains no sex chromosome mosaicism, so any misclassification would only affect the SCM group composition, not the control group purity. Given that our study was designed to detect clinically meaningful differences (a 10% absolute reduction in live birth rate) and found none, even substantial misclassification would be unlikely to fully explain the observed null effect.

Furthermore, tissue-specific mosaicism represents a well-documented phenomenon in sex chromosome disorders. Studies in Turner syndrome patients have demonstrated that mosaicism ratios can differ substantially between peripheral blood and oral epithelial cells (39), with cryptic mosaicism detected by FISH in approximately 25% of patients previously diagnosed with homogeneous monosomy based on standard cytogenetics alone (40). In the context of reproductive outcomes, gonadal or endometrial mosaicism—rather than peripheral blood mosaicism—would be the biologically relevant tissue for ART outcomes, as these tissues directly influence gametogenesis, embryo implantation, and early placental development. However, direct analysis of gonadal or endometrial tissue is invasive and not ethically feasible in asymptomatic infertile patients undergoing routine ART evaluation. Therefore, our reliance on peripheral blood karyotyping represents a surrogate marker that may not accurately reflect the mosaic status of reproductive tissues. This limitation is inherent to all studies using blood-based cytogenetic screening and highlights the urgent need for non-invasive or minimally invasive methods to assess tissue-specific mosaicism in reproductive organs.

### 4.7 Future research directions

Future research should focus on several critical directions: (1) multicenter prospective validation to confirm our findings in diverse populations; (2) single-cell sequencing of oocytes and ovarian tissue to elucidate the relationship between tissue-specific mosaicism and reproductive outcomes; (3) establishment of offspring follow-up cohorts to evaluate long-term health outcomes; (4) investigation of XCI skewing patterns as potential biomarkers for ovarian reserve and ART outcomes; (5) randomized controlled trials comparing stimulation protocols in mosaic patients; and (6) preimplantation genetic testing for aneuploidy (PGT-A) assessment of embryo aneuploidy rates in mosaic women to inform counseling regarding offspring risk.

## 5 Conclusion

This 1:4 propensity score-matched study demonstrates that low-level sex chromosome mosaicism (<10%) does not significantly affect live birth rate, clinical pregnancy rate, or early miscarriage rate in the first fresh ART transfer cycle. Female age remains the independent dominant predictor of ART outcomes. Age-stratified analyses revealed no significant mosaicism effects across all subgroups, with no synergistic negative interaction between female age and mosaicism status. These findings suggest that the reproductive prognosis for women with low-level sex chromosome mosaicism is comparable to that of chromosomally normal populations, providing important evidence-based guidance for genetic counseling and fertility management. Prospective multicenter studies are warranted for further validation.

## Data Availability

The datasets presented in this study can be found in online repositories. The names of the repository/repositories and accession number(s) can be found in the article/supplementary material.
